# Commentary: clonal hematopoiesis and breast cancer risk – new insights from the UK Biobank

**DOI:** 10.1097/JS9.0000000000003211

**Published:** 2025-08-20

**Authors:** Shengjie Wang, Bin Chen, Xiaozhen Zhan

**Affiliations:** Department of Thyroid and Breast Surgery, Xiamen Humanity Hospital, Fujian Medical University, Xiamen, China

*Dear Editor,*

We read with great interest the recent article[1] titled “*Clonal hematopoiesis of indeterminate potential and the risk of breast cancer: a UK Biobank cohort study*.” This large-scale prospective analysis provides timely and valuable insights into the relationship between clonal hematopoiesis of indeterminate potential (CHIP) and breast cancer risk. The integration of whole-exome sequencing (WES) from over 150 000 participants with detailed phenotypic and lifestyle data represent a significant contribution to cancer genomics and epidemiology.

The study systematically examined the association between CHIP and breast cancer incidence using both logistic regression and Cox proportional hazards models. After adjusting for key confounders, including body mass index (BMI), lifestyle factors, inflammatory markers, and polygenic risk scores (PRS), CHIP was associated with a 19% increased risk of breast cancer (adjusted OR = 1.19, *P* = 1.77e-04). These findings were consistently validated through stratified and sensitivity analyses, including exclusion of high-BMI individuals and those diagnosed shortly after enrollment. Furthermore, CHIP carriers demonstrated significantly higher cumulative incidence of breast cancer (Log-Rank *P* = 5.19e-05), underscoring its potential clinical relevance.

A notable strength of this study is its gene-specific analysis, identifying mutations in ATM (OR = 3.97) and DNMT3A (OR = 1.33) as key contributors to breast cancer risk. These results suggest the potential utility of incorporating CHIP mutational profiles into individualized risk prediction models and cancer prevention strategies.

We particularly commend the authors for their rigorous analytic framework, including the use of variant allele frequency (VAF) and extensive sensitivity analyses, which bolster the credibility of the findings. To further enhance this important work, we would like to offer several suggestions for future research:

First, while genetic predisposition was accounted for using a PRS, further clarification regarding the construction of the PRS would be valuable. Specifically, whether high-risk susceptibility genes such as BRCA1, BRCA2, PALB2, and CHEK2 were incorporated or modeled separately remains unclear. Given their established role in hereditary breast cancer, this information is crucial for accurate genetic risk estimation and the interpretation of CHIP-related risk.

Second, although the multivariable models adjusted for several established breast cancer risk factors, some clinically important variables – such as menopausal status, hormone replacement therapy (HRT) use, and family history – were not explicitly reported. Their omission may introduce residual confounding.

In addition, the impact of CHIP across distinct molecular subtypes of breast cancer (e.g., ER +, HER2 +, triple-negative) and its potential influence on treatment response were not extensively explored. Future studies integrating tumor subtype information and treatment outcomes could help determine whether CHIP-related mutations have subtype-specific prognostic or predictive value.

Finally, incorporating multi-omics data – including transcriptomics, epigenomics, and proteomics – may reveal key regulatory pathways linking CHIP to breast carcinogenesis. Such integrative analyses can facilitate biomarker discovery and support precision prevention strategies.

In summary, this study marks an important step in elucidating the role of CHIP beyond hematologic malignancies. The identification of ATM and DNMT3A as high-impact mutations may hold particular relevance for early detection and risk stratification in breast cancer. We thank the authors for this important contribution and look forward to future research that expands upon these findings.

## Data Availability

All data analyzed or generated during this study are included in the article.
